# *Desulfohalophilus alkaliarsenatis* gen. nov., sp. nov., an extremely halophilic sulfate- and arsenate-respiring bacterium from Searles Lake, California

**DOI:** 10.1007/s00792-012-0468-6

**Published:** 2012-06-29

**Authors:** Jodi Switzer Blum, Thomas R. Kulp, Sukkyun Han, Brian Lanoil, Chad W. Saltikov, John F. Stolz, Laurence G. Miller, Ronald S. Oremland

**Affiliations:** 1 US Geological Survey, Menlo Park, CA 94025 USA; 2 Department of Geological Sciences and Environmental Studies, Binghamton University, Binghamton, NY 13902 USA; 3 Department of Biological Sciences, University of Alberta, Edmonton, AB Canada; 4 Department of Microbiology and Toxicology, University of California, Santa Cruz, CA 95064 USA; 5 Department of Biological Sciences, Duquesne University, Pittsburgh, PA 15282 USA

**Keywords:** Alkaliphile ecology, Systematics, Anaerobic bacteria, Metaloxidation and reduction, Halophiles, Ecology, Biotechnology, Phylogeny, Genetics, Taxonomy, Enzymes

## Abstract

A haloalkaliphilic sulfate-respiring bacterium, strain SLSR-1, was isolated from a lactate-fed stable enrichment culture originally obtained from the extreme environment of Searles Lake, California. The isolate proved capable of growth via sulfate-reduction over a broad range of salinities (125–330 g/L), although growth was slowest at salt-saturation. Strain SLSR-1 was also capable of growth via dissimilatory arsenate-reduction and displayed an even broader range of salinity tolerance (50–330 g/L) when grown under these conditions. Strain SLSR-1 could also grow via dissimilatory nitrate reduction to ammonia. Growth experiments in the presence of high borate concentrations indicated a greater sensitivity of sulfate-reduction than arsenate-respiration to this naturally abundant anion in Searles Lake. Strain SLSR-1 contained genes involved in both sulfate-reduction (*dsrAB*) and arsenate respiration (*arrA*). Amplicons of 16S rRNA gene sequences obtained from DNA extracted from Searles Lake sediment revealed the presence of close relatives of strain SLSR-1 as part of the flora of this ecosystem despite the fact that sulfate-reduction activity could not be detected in situ. We conclude that strain SLSR-1 can only achieve growth via arsenate-reduction under the current chemical conditions prevalent at Searles Lake. Strain SLSR-1 is a deltaproteobacterium in the family Desulfohalobiacea of anaerobic, haloalkaliphilic bacteria, for which we propose the name *Desulfohalophilus alkaliarsenatis* gen. nov., sp. nov.

## Introduction

Environments characterized as “extremes” of temperature, pH, salinity, radiation, aridity, toxicants, or combinations of these factors have long held fascination to biologists. While some hypersaline environments can provide habitat to a selected few species of well-adapted metazoans (e.g., *Artemia*), they can also harbor dense and diverse populations of microorganisms (e.g., Bodaker et al. 2010). Yet even within the context of salinity extremes, there are gradations. The term “hyper-saline” is loosely defined as exceeding that of oceanic salt strength (~35 g/L). The upper range of hyper-salinity implies brines with high specific gravities (and low water activities); hence values of dissolved salts would significantly exceed 150 g/L and range up to and including dense, salt-saturated brines (~350 g/L). It is at this upper edge of extreme hyper-salinity that even microbial life becomes challenged. The Bacteria and Archaea that manage to inhabit dense brines are particularly adapted to tolerate low water activities by a variety of evolved molecular strategies to cope with osmotic stress. For anaerobes, which have an even more constrained bio-energetic regimen than aerobes, these physical challenges are even more difficult to bear (Oren 1999a, b).

Amongst the anaerobes, there is considerable scientific interest in sulfate-respiring prokaryotes owing in part to the ubiquity of these microbes in anoxic environments and the abundance of sulfate anions in saline systems, especially seawater (~28 mM). The biogeochemical process of dissimilatory sulfate-reduction is thought to be ancient, deriving from Archean times when the global oceans were much less saline and held much less sulfate content than today (Shen et al. 2001). Over the past 3.5 billion years, however, many sulfate-rich hyper-saline brines have appeared both on the Earth’s surface and buried within its crust. Oren (1999a) made the empirical observation that dissimilatory sulfate-reduction in natural waters may be constrained at only the very upper reaches of salinity, essentially reaching a cap at salt saturation (≥300 g/L), although no known pure cultures could achieve growth at the elevated salt levels that approached saturation. Hence, water bodies that are moderately hypersaline, such as Solar Lake (~150 g/L), Big Soda Lake (27–89 g/L), and Mono Lake (~90 g/L) have high primary productivity coupled with intensive sulfate-reduction (Cohen et al. 1977; Jørgensen and Cohen 1977; Cloern et al. 1983; Smith and Oremland 1987; Jellison and Melack 1993; Oremland et al. 2000). However, within sulfate-containing brines that are poised at salt saturation, clear evidence for the occurrence of bacterial sulfate-reduction is somewhat ambiguous. For example, in the case of the Dead Sea, the stable sulfur isotopic work of Nissenbaum and Kaplan (1976) was cited by Oren (1999a) as evidence for in situ sulfate-reduction. Nonetheless, direct evidence of sulfate reduction based on the use of ^35^S-radiotracer in Dead Sea sediments is lacking, although not for a want of trying (Oren 1999b). Similarly, sediment profiles of dissolved constituents in the Orca Brine suggested that the extreme salinity therein completely inhibited sulfate-reduction (Weisenburg et al. 1985). Contrasting with this is the report of active ^35^S-sulfate-reduction within the dense brines at the bottom of the eastern Mediterranean Sea (van den Wielen et al. 2005) and the amplification of dissimilatory sulfite reductase gene sequences (*dsrA*) in samples taken from the L’Atalante and Urania basins of those brines (van den Wielen and Heijs 2007).

The seminal review by Oren (1999a) proposing an upper salinity limit for sulfate reduction and other terminal electron accepting processes (such as methanogenesis) prompted others to more closely examine water bodies poised at the upper reaches of salinity for, amongst other microbial processes, the presence of detectable sulfate-reduction. Thus, ^35^S-sulfate-reduction was shown to be active in the moderately hyper-saline southern arm of the Great Salt Lake (salinity ~120 g/L) and was about tenfold lower but still detectable in the much more saline (~270 g/L) but still unsaturated northern arm (Kjeldsen et al. 2007). Sulfate-reducing bacteria could be both cultured as well as detected by culture-independent means (i.e., 16S rRNA and dissimilatory sulfite reductase, *dsrAB*, gene sequences) from the sediments of both arms of the Great Salt Lake (Brandt et al. 2001; Jakobsen et al. 2006; Kjeldsen et al. 2007). In a survey of ^35^S-sulfate-reduction assayed in sediments over salt concentration gradients in a series of coastal salt pans in South Africa, the highest detected rates were actually reported at the highest measured salinities, including super-saturation (e.g., 422 g/L; Porter et al. 2007). These results contrasted with an earlier survey conducted along South San Francisco Bay evaporation ponds which found greatly diminished activity at the highest salinities (Klug et al. 1985). The aforementioned investigations were performed in very saline water bodies that were poised at circum-neutral pH (~6.0–8.2). In a broad survey of hyper-saline and highly alkaline soda lake sediments located in Siberia, some of the highest ^35^S-sulfate-reduction rates occurred at the highest measured salinities, including super-saturation (e.g., 475 and 520 g/L), and the presence of sulfate-reducing bacteria in these systems was also confirmed by both culture-dependent and culture-independent techniques (Foti et al. 2007). These authors also had success in cultivating enrichment cultures of sulfate-reducers in a medium containing 4 M Na_2_CO_3_ (~424 g/L) with a mixture of lactate and butyrate serving as electron donors. Aspects of these above given efforts that transpired over the past decade were recently summarized by Oren (2011).

Enigmatically, in stark contrast to the above reports stand our results for Searles Lake, the alkaline salt-saturated brine located in the Mojave Desert of California. This lake is particularly rich in arsenic oxyanions (~4 mM) along with other toxic elements, most notably boron (0.46 molal; roughly equivalent to 0.62 M) (Oremland et al. 2005). Although the sediments contained some detectable free sulfide (~0.1 mM), all attempts to elicit ^35^S-sulfate-reduction from them failed (Kulp et al. 2006), including manipulations of incubated slurries designed to enhance activity by such stratagems as amendment with exogenous electron donors (i.e., lactate or H_2_), by lowering the sulfate-content of artificial brine matrix, or by raising the specific activity of the isotope applied (Kulp et al. 2007). We eventually were able to point to borate ions acting as direct inhibitors of sulfate-reduction as the primary reason for the absence of detectable sulfate-reduction in this system. Our subsequent investigations on the ecophysiology of the arsenate-respiring bacterium *Halarsenatibacter silvermanii* strain SLAS-1 isolated from this system clearly demonstrated the occurrence of sulfate-reduction within a stable, lactate-fed enrichment culture that included strain SLAS-1. Yet this sulfate-reducer was a separate, distinct entity from strain SLAS-1 (Switzer Blum et al. 2009). We now report isolation from this enrichment culture of *Desulfohalophilus alkaliarsenatis* strain SLSR-1; an isolate that is capable of sustaining anaerobic respiratory growth at near salt-saturation (~330 g/L), equivalent to a water activity (*a*_w_) of 0.81. The significance of this finding with regard to the search for microbial life beyond Earth will be discussed.

## Materials and methods

### Enrichment culture and isolation

Strain SLSR-1 was isolated from a Searles Lake enrichment culture exhibiting clear evidence for the presence of sulfate-reduction, as determined by increases in sulfide with growth, and verified by the reduction of ^35^S-sulfate to ^35^S-sulfide (Switzer Blum et al. 2009). After isolation of strain SLAS-1, efforts were then re-oriented to isolate an extremely halophilic sulfate-reducer using the same anaerobic, high-salt lactate-based SearlesAb1 medium (salinity 340 g/L) described in Switzer Blum et al. (2009) but lacking any added arsenate, and thereby having sulfate as the sole electron acceptor. The composition of SearlesAb1 basal salts was (g/L): NaCl (180), Na_2_SO_4_ (100), K_2_SO_4_ (30), Na_2_CO_3_ (27), NaHCO_3_ (5.0), H_3_BO_3_ (4.0), (NH_4_)_2_SO_4_ (0.05), KH_2_PO_4_ (0.08), K_2_HPO_4_ (0.15), MgSO_4_·7H_2_O (0.025), Na_2_WO_4_ (0.075), Na_2_SeO_4_ (0.00001), and 3.0 ml of SL10 trace element solution (Widdel et al. 1983), yeast extract (0.2), cysteine-HCl (0.25), and 10 ml of a vitamin mix (Wolin et al. 1963; Oremland et al. 1994). The pH was adjusted to 9.5 with NaOH. The water activity (*a*_w_) of SearlesAb1 medium was 0.8 as determined analytically (see below). After three successive transfers at 38 °C, a serial dilution was conducted, with the 10^−8^ dilution level still showing growth. However, a microorganism similar in its odd motility to that displayed by *Halarsensatibacter silvermanii* strain SLAS-1 was also present (Switzer Blum et al. 2009). Hence, subsequent purification was achieved by conducting another dilution series at 20 °C, a temperature at which *H. silvermanii* strain SLAS-1 does not grow. In this case the 10^−2^ dilution demonstrated only one morphological type to be present. We were unable to cultivate this organism on solid agar plates composed of SearlesAb1 medium and thus could not pick isolated colonies. However, purity of the liquid cultures was confirmed by the presence of only a single band upon DGGE analysis of amplified 16S rRNA genes (see below) and designated strain SLSR-1. We subsequently determined that strain SLSR-1 did not require yeast extract for growth. Hence, other than in cases specified below to enhance growth rates, we eliminated it from the medium.

### Growth experiments

The SearlesAb1 medium described above was used for growth experiments but modified with respect to electron acceptors added (sulfate or arsenate or both), the amount of electron donor (lactate) used, and the overall salinity employed. The salinity was lowered to 157 g/L (*a*_w_ = 0.91) to improve growth. This was achieved by decreasing the amounts of NaCl (125 g/L), NaHCO_3_ (4 g/L), and Na_2_CO_3_ (24 g/L). Sulfate concentrations were manipulated for various media either by its total elimination or by adding incremental amounts of Na_2_SO_4_ (0.6–6 g/L), and K_2_SO_4_ (0.2–1.0 g/L). In order to be able to analytically discern lactate consumption in the presence of limited electron acceptor, we varied the amount of lactate employed. High concentrations (~60 mM) were used when conducting growth experiments with sulfate (30 mM), but lower concentrations of 15 mM lactate were used for sulfate (5 mM) plus arsenate (7 mM), and 3 mM lactate for arsenate alone (5 mM).

### Effects of salinity, temperature, pH, and borate anions upon growth

The salinity range for growth was determined by varying the amount of NaCl added to SearlesAb1 medium (range 9–284 g/L). This was done individually for growth on sulfate and arsenate. Because growth in the basal salts medium was so slow during the above described salinity range experiments, the medium was modified by addition of 0.2 g/L of yeast extract for the pH, temperature, and borate concentration range experiments, which allowed us to shorten growth incubations from several months to several weeks. The pH range for growth was determined by employing the SearlesAb1 medium set at a salinity of 296 g/L (NaCl = 150 g/L), and the pH varied by changing the amounts of carbonate versus bicarbonate in the medium as previously described (Switzer Blum et al. 1998). The pH was re-checked in all tubes after the incubations and no deviations from the initial set of pH determinations occurred. Identical medium was employed for the temperature range experiments. The medium used to determine the effects of added borate anions upon growth under sulfate- or arsenate-reducing conditions was composed of SearlesAb1 as modified for a salinity of 180 g/L and a range of added borate (0–300 mM) and the pH was maintained at 8.8. In these long-term incubations (2–3 months) we used sulfide production or arsenate reduction in lieu of cell counts as proxies for cell growth

### Experiments with washed cell suspensions

For the purpose of determining whether strain SLSR-1 achieved respiratory growth on nitrate by denitrification or by dissimilatory reduction to ammonia, we conducted incubations with washed cells suspensions. Cells were grown in lactate + nitrate Searles Ab1 medium (salinity 200 g/L), harvested by centrifugation, washed three times with a pH 9.5 buffer solution (g/L): NaCl (142), KH_2_PO_4_ (0.08), K_2_HPO_4_ (0.15), MgSO_4_·7H_2_O (0.025), H_3_BO_3_ (0.3), Na_2_CO_3_ (24), NaHCO_3_ (8.0), K_2_SO_4_ (0.87). All manipulations were carried out in an anaerobic glove box. Cells were re-suspended in buffer and supplemented with 15 mM Na lactate and 10 mM NaNO_3_ and 0.25 % cysteine-HCl as reducing agent and dispensed in 10 mL portions into serum bottles (37 mL volume) and crimp-sealed with butyl rubber stoppers and flushed with oxygen-free N_2_. Bottles were incubated statically at 38 °C, and selected samples were amended with a 15 % headspace addition of acetylene gas to inhibit N_2_O reductase (Balderston et al. 1976) using ^63^Ni electron-capture gas chromatography to detect headspace N_2_O and chemical analysis of liquid phase subsamples for their ammonia content (see below).

### Analytical

Herein we have attempted to introduce the usage of water activity (*a*_w_) as a supplemental parameter to describe the experimental physiological constraints faced by extreme halophiles (Table 1). Activity of water (*a*_w_) was determined on stored samples and media using an Aqualab Water Activity Meter, Pawkit system (Decagon Devices, Pullman, WA). This device measures the humidity of an equilibrated enclosed air volume lying above the aqueous sample. Precision of triplicate measurements was 0.01 and accuracy was 0.02. Water activity measurements were conducted on SearlesAb1 medium, as well as on previously collected and archived (refrigerated at 6 °C) samples from Searles Lake, Mono Lake, and the Dead Sea. Organic acids, arsenic oxyanions, and sulfate were determined by high performance liquid chromatography (Hoeft et al. 2004), sulfide by the spectrophotometric method of Cline (1969), and direct cell counts by acridine orange staining (Hobbie et al. 1977). The acetylene-block technique of N_2_O-reductase (Balderston et al. 1976; Oremland et al. 1984) was used as a presumptive test for the presence of a full denitrification pathway leading to N_2_ formation from nitrate reduction. Ammonia in the aqueous phase was determined by the method of Solorzano (1969). Membrane-associated fatty acids (PLFAs: phospho-lipid fatty acids), a commonly used taxonomic tool, were obtained by harvesting lactate + sulfate-grown cells (Searles Ab1 medium; salinity 292 g/L), and the centrifuged frozen pellet was shipped to Microbial ID Inc. (Newark, DE). Analyses were performed on fatty acid methyl esters of the extracted PLFAs by flame ionization gas chromatography by methods detailed on-line (http://www.microbialid.com).

**Table 1:** Estimated salinity composition of artificial media and natural brines compared with water activity measurements

Component	Salinity (g/L)	Water activity (*a*_w_)
Searles Lake brine	>350	0.71
Searles Lake medium	346	0.79
Searles Lake medium	330	0.81
Searles Lake medium	296	0.83
Searles Lake medium	280	0.84
Searles Lake medium	230	0.87
Searles Lake medium	180	0.89
Searles Lake medium	160	0.91
Searles Lake medium	130	0.92
Searles Lake medium	80	0.95
Searles Lake medium	54	0.96
Mono Lake brine	~80	0.92
Mono Lake medium (AML60)^a^	75	0.94
Dead Sea brine	~337	0.67
Dead Sea medium (high)^b^	325	0.78
Dead Sea medium (low)	205	0.89

^a^Switzer Blum et al. (1998)

^b^Switzer Blum et al. (2001)

### TEM/SEM

Sample preparation, thin section micro-toning, and scanning/transmission electron microscopy were carried out by procedures previously described (Switzer Blum et al. 2009).

### Molecular analysis and alignments of strain SLSR-1 16S rRNA, *dsrA*, and *arrA* genes

DNA was extracted from a culture of SLSR-1 as previously described (Kulp et al. 2007). 16S rRNA genes and functional gene markers for dissimilatory sulfite reduction (*dsrAB*) were PCR amplified, cloned, screened, sequenced, and aligned as described previously (Kulp et al. 2007; Switzer Blum et al. 2009; Hoeft et al. 2010). For 16S rRNA and *dsrAB* genes, their evolutionary history was inferred using either the neighbor-joining method (16S rRNA genes; Saitou and Nei 1987) or the maximum likelihood method (*dsrAB* genes; Schwarz and Dayhoff 1979). The bootstrap consensus 16S rRNA gene tree inferred from 500 replicates was taken to represent the evolutionary history of the taxa analyzed for both 16S rRNA and *dsrAB* genes (Felsenstein 1985), and branches corresponding to partitions reproduced in <50 % bootstrap replicates were collapsed. The percentage of replicate trees in which the associated taxa clustered together in the bootstrap test (500 replicates) were aligned next to the branches displayed. The 16S rRNA gene tree was drawn to scale, with branch lengths in the same units as those of the evolutionary distances used to infer the phylogenetic tree. The evolutionary distances were computed using the 2-parameter method (Kimura 1980) and were given in the units of the number of base substitutions per site. The analysis involved 40 nucleotide sequences. All positions containing gaps and missing data were eliminated. There were a total of 490 positions in the final dataset, and evolutionary analyses were conducted in MEGA5 (Tamura et al. 2007). For the *dsrAB* genes, the initial tree(s) for the heuristic search were obtained automatically as follows: When the number of common sites was <100 or less than one fourth of the total number of sites, the maximum parsimony method was used; otherwise, BIONJ method with MCL distance matrix was used. The tree was drawn to scale, with branch lengths measured in the number of substitutions per site. The analysis involved 21 amino acid sequences. All positions containing gaps and missing data were eliminated. There were a total of 502 positions in the final dataset. Evolutionary analyses were conducted in MEGA5 (Tamura et al. 2007).

The initial amplicon using an *arrA* primer set generated a ~200-bp product that was used to sequence flanking DNA by an inverse PCR approach as described in Switzer Blum et al. (2009) and Saltikov and Newman (2003). Inverse primers (SLSR1-iPCR-F1, 5′-CCT GGT CAA ATG GTG GAA T-3′ and SLSR1-iPCR-R1 5′-GCT CCG TCC TTG AAG TCT C-3′) were used in PCRs on *Eco*RI or *Hin*dIII digested and circularized SLSR-1 genomic DNA. The PCR amplicons were cloned, sequenced, and assembled into a ~1.8-kb DNA fragment. Phylogenetic analysis was performed on the translated ArrA of the partial *arrA* gene sequences from SLSR-1 and other bacteria as previously described (Switzer Blum et al. 2009; Zargar et al. 2012). Sequences were aligned using ClustalW (Thompson et al. 1994). PAUP* (Swofford 1999) was used to generate a neighbor-joining phylogeny on the resulting multi-sequence alignment where gaps in the alignment were ignored.

### Nucleotide sequence accession numbers

The nucleotide sequences of 16S rRNA gene, *dsrAB*, and *arrA* genes of SLSR-1 submitted to GenBank were given the accession numbers JQ582408, JQ 582409, and JQ955737, respectively.

### G + C content of strain SLSR-1

The molar G + C content of strain SLSR-1 was obtained from frozen cells shipped to the DSMZ using their standard extraction protocol as delineated elsewhere (http://www.dsmz.de/services/services-microorganisms/identification/gc-content.html).

### Molecular analysis of 16S rRNA genes in Searles Lake sediments

16S rRNA gene sequences related to strain SLSR-1 were detected by DGGE of samples of Searles Lake sediment collected in 2005 (Kulp et al. 2006). To confirm relatedness to strain SLSR-1, we excised bands from Searles Lake 16S rRNA gene assemblages that migrated to the same position as strain SLSR-1 and sequenced them to confirm similarity as previously described (Kulp et al. 2007). Sequences with >97 % 16S rRNA gene similarities to strain SLSR-1 were considered to be highly related.

## Results

### Water activity measurements (*a*_w_)

The *a*_w_ values for three naturally occurring brines and for our artificial media are shown in Table 1. Mono Lake surface water and our standard cultivation medium for that environment (AML60) were nearly identical and comparable to the values for Searles Lake media with the lowest applied salts. Searles Lake high salinity medium (346 g/L) yielded a much lower value of *a*_w_ (0.79), but it was still higher than the natural brine sample from that environment (~0.71). By comparison, an archived Dead Sea sample exhibited the lowest value of all the natural brines and artificial media tested (~0.67), an *a*_w_ that can only be achieved in brines containing divalent cations such as Ca^2+^ or Mg^2+^.

### Morphological characteristics of strain SLSR-1

**Fig. 1:**
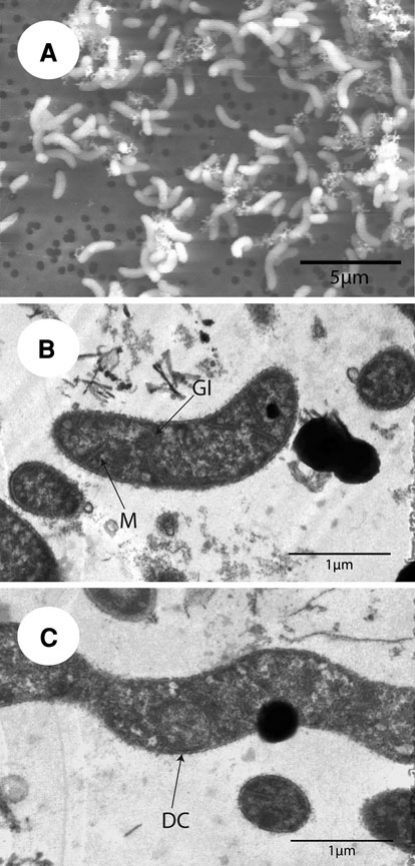
Electron micrographs of strain SLSR-1. **a** Scanning electron micrograph of sulfate-grown cells. **b** Thin section transmission micrograph of sulfate-grown cells showing presence of internal membranes (*M*-*labeled arrow*) and granular inclusions (*G-labeled arrow*). **c** Thin section of arsenate-grown cells showing presence of an internal daughter cell located near the node between larger cells (*D-labeled arrow*)

**Fig. 2:**
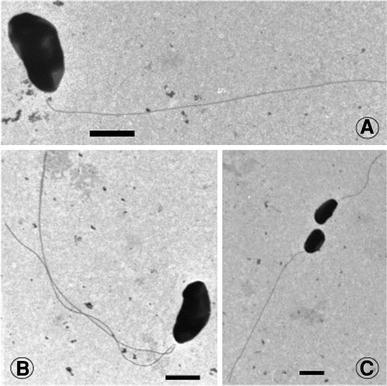
Transmission electron micrographs showing the polar flagella of strain SLSR-1, which most commonly were observed singly (**a**, **c**), but sometimes were paired (**b**). *Bars* indicate 1 μm

Strain SLSR-1 cells are motile vibrios (3.0 × 0.5 μm) (Fig. 1a) that stain Gram-negative. Thin sections exhibited numerous granular inclusions as well a system of internal membranes (Fig. 1b). In some instances fully formed, coccoid daughter cells having their own double outer membranes were observed within the structure of their parent cells (Fig. 1c). Transmission electron microscopy revealed that motility was conferred by a long (~10 μm) polar flagellum (Fig. 2), usually observed as a single array (Fig. 2a, c), but in rare instances, as paired polar flagella (Fig. 2b). The flagella of strain SLSR-1 proved highly fragile during the TEM processing, and many simply broke off the samples, leaving behind unadorned cells.

### Growth characteristics of strain SLSR-1

**Fig. 3:**
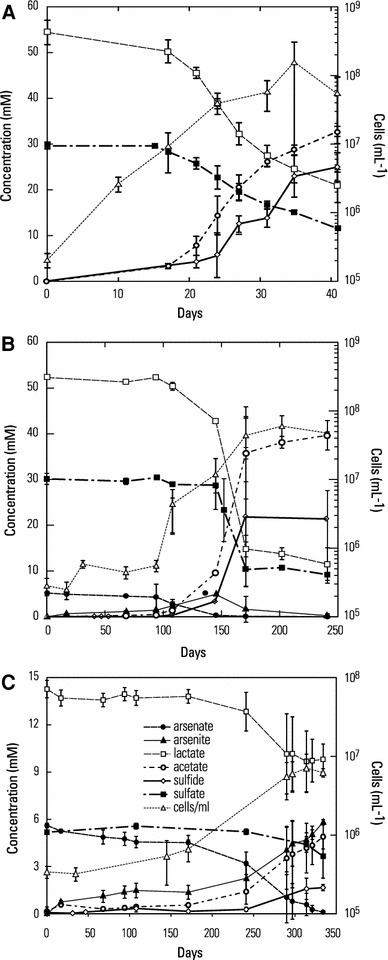
Growth curves of strain SLSR-1 achieved on defined media (salinity 157 g/L; *a*_w_ = 0.91) with lactate as the electron donor. **a** Growth on high concentrations of sulfate (30 mM) and lactate (50 mM). **b** Growth on high concentrations of sulfate and lactate plus 5 mM arsenate. **c** Growth on lower concentrations of sulfate plus arsenate (~6 mM) with 15 mM lactate. Symbols represent the mean of three cultures. *Bars* indicate +1SD

Cell growth was evident on a lactate/sulfate medium with consumption of both over time, concomitant production of acetate and sulfide, and a cell density increase of 2 orders of magnitude, reaching a maximum (~1 × 10^8^ cells/ml) by 36 days (Fig. 3a). There was a stoichiometric balance between lactate consumed (~33 mM) and acetate produced (~32 mM) indicating a decarboxylation with the production of CO_2_. The relationship between sulfate consumed (~18 mM) and sulfide formed (~25 mM), however, was only in very rough balance. Growth was slow (doubling time 82.5 h; μ = 0.008 h^−1^) at this salinity (160 g/L; *a*_w_ = 0.9). In medium containing 5 mM As(V) but otherwise identical to the previous one (sulfate 30 mM), about 100 days elapsed before exponential growth was initiated, at which time As(V) reduction to As(III) briefly preceded sulfate reduction (Fig. 3b). There was reasonably good agreement between lactate and sulfate consumed, 43 and 18 mM, respectively, and the final accumulated amounts of acetate (40 mM) and sulfide (22 mM) produced. Arsenate was undetectable by ~140 days and was entirely replaced by As(III), which was also eventually removed, presumably as thioarsenite as a result of reaction with free sulfide in the medium. When sulfate and As(V) were both present at equivalent initial concentrations (5 mM) and the experiment conducted with much lower lactate (15 mM), a slow rate of growth and of As(V) reduction to As(III) occurred for the first 175 days, after which logarithmic phase was attained (Fig. 3c). Under these conditions, As(V) reduction clearly preceded that of sulfate-reduction, and the latter process did not commence until all the As(V) was reduced to As(III). By the incubation’s end there was a consumption of 5.75 mM lactate, which would have generated 23 mmol equiv. electrons. This source was balanced by the observed reduction of all the 5 mM As(V) plus 1.75 mM SO_4_^2−^, thereby accounting for 24 mmol equiv. electrons as a cumulative respiratory sink. No growth or sulfate-reduction occurred in killed controls, or live samples incubated with lactate but without sulfate (data not shown). We did not observe growth when arsenate was employed as the sole electron acceptor unless some sulfate (~1 mM) was present in the medium (not shown).

### Effects of salinity, pH, temperature, and borate

**Fig. 4:**
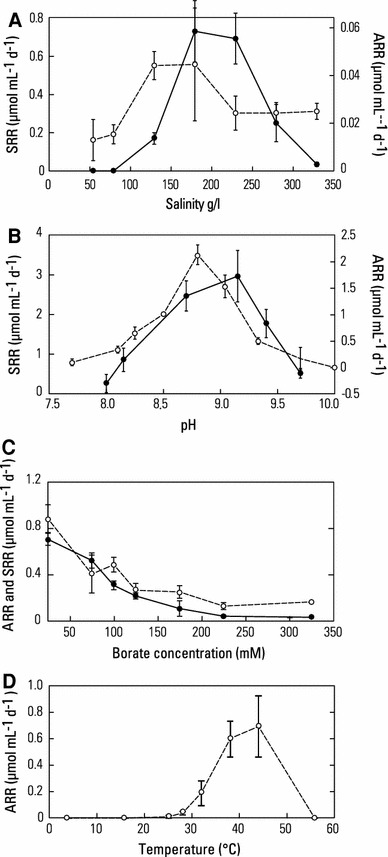
Physiological characterizations of strain SLSR-1 grown on either sulfate (*filled circles*) or arsenate (*open circles*). **a** Salinity. **b** pH. **c** Borate. **d** Temperature. Symbols represent the mean of three cultures and *bars* indicate +1SD. The medium for the pH, borate, and temperature experiments was supplemented with 0.2 % yeast extract to improve growth rates

The salinity range for growth on sulfate was broad (125–330 g/L; *a*_w_ = 0.93–0.8) with an optimum of ~200 g/L or *a*_w_ = 0.84 (Fig. 4a). Curiously, the range for arsenate was even greater, with activity evident as low as 55 g/L (*a*_w_ = 0.96), and an optimum between 125 and 175 g/L (*a*_w_ = 0.92–0.89). Because we assumed a similar physiological response to pH with regard to available electron acceptor, we ran these salinity experiments at an alkaline pH of 9.5. However, an unexpected finding was that the pH optimum for growth on sulfate (~9.25) was higher than that for arsenate (8.75) (Fig. 4b). The response to borate additions is shown in Fig. 4c. Borate proved inhibitory to both sulfate reduction and arsenate reduction, but the former proved more sensitive and was completely blocked at 200 mM BO_3_^3−^. In contrast, arsenate reduction was still evident at that concentration, and it was still in evidence at 300 mM BO_3_^3−^, although it was strongly impaired (~78 % inhibition). The temperature range for growth was 20–50 °C, with an optimum at 44 °C and no growth evident at 16 °C, very slow growth at 20 °C, and no growth at 55 °C (Fig. 4d).

### Electron donors and acceptors

The substrate affinities and other phenotypic characterizations of strain SLSR-1 are listed in Table 2, and compared with those of 3 other species of anaerobic haloalkaliphiles capable of dissimilatory reduction of sulfate from the family Desulfohalobiaceae. Growth of strain SLSR-1 could be supported by the electron-donors lactate or pyruvate, but not by acetate, H_2_, H_2_ + acetate, formate or ethanol. Electron acceptors supporting growth on lactate besides sulfate and arsenate included nitrate, thiosulfate and sulfite, but not sulfur, fumarate, selenate, or Fe(III). In addition to heterotrophic growth on the listed organics, growth was also observed when sulfide was the e-donor and nitrate served as the e-acceptor. Growing cells consumed nitrate (~15.3 mM), produced sulfate (~4.0 mM), and traces of N_2_O were observed in the headspace of the culture tubes after several days’ incubation (69.6 ± 44.6 nmoles; *n* = 3). Growth could be sustained in this fashion upon repeated transfers, suggesting that growth was a result of chemolithoautotrophy and not the result of carry-over of an organic carbon source during inoculation. We also observed a capacity for oxidation of sulfide coupled to arsenate reduction, but growth could not be sustained in this fashion with repeated transfer. This implied a capacity for lithotrophy with the sulfide/arsenate coupling, but not for chemoautotrophic growth.

**Table 2:** Phenotypic characteristics that distinguish *Desulfohalophilus alkaliarsenatis* strain SLSR-1 from other halo-alkaliphilic sulfate- or sulfur-reducers isolated from soda lakes

Characteristic	SLSR-1	*Desulfonatronospira thiodismutans* ^a^	*Desulfohalobium retbaense* ^b^	*Desulfonatronovibrio hydrogenovorans* ^c^
Habitat	Searles Lake, CA, USA	Kulanda Steppe, Russia	Retba Lake, Senegal	Lake Magadi, Kenya
Morphology	Vibroid	Vibroid	Rod	Vibroid
Motility	+	+	+	+
Flagella	+	+	+	+
Size (μm)	0.5 × 3.0	0.6–0.8 × 2–30	0.7–0.9 × 1.0–3.0	0.5 × 1.5–2.0
pH range (optimum)	7.75–9.75 (9.25)	8.3–10.5 (10)	5.5–8.0 (6.5–7.0)	8.0–10.2 (9.6)
Salt range (g/L)	55–330	87–232	29–240	10–120
Temperature range (opt.; °C)	20–50 (44)	“Mesophilic” (43)	25–40 (37)	(37)
G + C content (mole%)	45.1	50.4	57.1	48.6
Chemolithotrophy				
H_2_	−	+	−	+^d^
S_2_^−^ + NO_3_^−^	+	ND^e^	ND	ND
S_2_^−^ + As(V)^d^	+	ND	ND	ND
Electron donors				
Lactate	+	+	+	−
Pyruvate	+	+	+	−
Acetate	−	−	−	−
H_2_ + acetate	−	−	+	+
Formate	−	+	+	−
Ethanol	−	+	+	−
Malate	−	ND	−	−
Succinate	−	ND	−	ND
Propionate	−	ND	−	−
Citrate	−	ND	ND	
Glycerol	−	ND	−	−
Glucose	−	ND	−	ND
Fructose	−	ND	−	ND
Fumarate	−	ND	−	−
Electron acceptors				
Sulfate	+	+	+	+
Arsenate	+	ND	ND	ND
Nitrate	+	ND	ND	ND
Nitrite	−	ND	ND	ND
DMSO	−	ND	ND	
Sulfite; thiosulfate	+	+^f^	+	+^f^
Oxygen	−	−	ND	
Sulfur	−	ND	+	−
Fumarate	−	ND	ND	ND
Fe(III)	−	ND	ND	ND
Mn(IV)	−	ND	ND	ND
Selenate	−	ND	ND	ND

^a^Sorokin et al. (2008)

^b^Ollivier et al. (1991)

^c^Zhilina et al. (1997)

^d^Lithotrophic growth required the presence of lactate or acetate

^e^ND = not determined

^f^Disproportionation of thiosulfate and/or sulfite is also possible

Incubation of washed cells with ~10 mM nitrate plus lactate produced N_2_O at a linear rate over a month-long incubation (data not shown) and accumulated 35.7 ± 2.4 nmoles N_2_O in the headspace by that time, which represented only ~0.07 % of the available nitrate. Samples (*n* = 3) incubated with C_2_H_2_ produced N_2_O, but at a ~fourfold slower rate than controls and accumulated only 10.6 ± 3.1 nmoles N_2_O in the headspace. During denitrification much more N_2_O would be expected in the presence of acetylene; hence these results indicated the lack of a functional N_2_O reductase. When compared with accumulated NH_4_^+^, N_2_O was only a minor product. Samples incubated without acetylene accumulated more NH_4_^+^ (27.1 ± 1.4 μmoles) by the end of the incubation than those incubated in the presence of acetylene (14.1 ± 1.1 μmoles). By the end of the incubation, cells had consumed 52 μmoles nitrate and accumulated 14 μmoles nitrite plus 27 μmoles NH_4_^+^ as products, thereby accounting for 79 % of the nitrate removed, with the 21 % difference probably accountable via assimilatory metabolic pathways. Acetylene-inhibited samples by comparison demonstrated only 36 μmoles nitrate consumed, and were thus impaired by ~31 % in this regard, suggesting acetylene partially inhibited the nitrate reductase of strain SLSR-1. These results show that nitrate respiration by strain SLSR-1 proceeded via dissimilatory reduction to ammonia.

### Phospho-lipid fatty acids

The major PLFAs, accounting for all those found in strain SLSR-1, can be seen in Table 3. Of the four molecular types determined, the group comprising normal, saturated fatty acids was the most prominent (44.8 %), with carbon chain lengths of 12, 16, and 18, respectively, having 6.7, 13.0, and 13.7 % abundances. There was one prominent (14 %) branched, saturated PLFA (15:0 iso) that was also a major PLFA found occurring in the haloalkaliphiles *Desulfonatronospira thiodismutans* and *Dspira. delicata* (Sorokin et al. 2008), but the other major PLFAs determined for these two species are different from those found in strain SLSR-1. Similarly, this (15:0 iso) branched PLFA occurs in *Desulfonatronovibrio magnus* but not in *Dvibrio. thiodismutans* (Sorokin et al. 2011a, b). *Dvibio. magnus* also contains 16:0 as a major normal, saturated fatty acid which is found in abundance in strain SLSR-1 (see above). *Halarsenatibacter silvermanii* strain SLAS-1 also contained abundant C16:0 and C18:0 as well as 15:0 iso PLFAs (Switzer Blum et al. 2009)

**Table 3:** Major membrane fatty acid (PLFA) composition of strain SLSR-1

Fatty Acid^a^	Percent
Branched/saturated	
11:0 iso	3.4
13:0 iso	3.2
15:0 iso	14.0
15:0 anteiso	0.3
15:0 iso DMA	3.0
17:0 iso	3.9
17:0 anteiso	1.7
	Total = 29.5 %
Branched/unsaturated	
15:1 iso w9c	0.8
	Total = 0.8 %
Normal/saturated	
12:0	6.7
12:0 DMA	0.5
14:0	1.4
14:0 DMA	0.7
16:0	13.0
16:0 DMA	2.8
16:0 aldehyde	1.0
17:0	1.5
18:0	13.7
18:0 DMA	3.5
	Total = 44.8 %
Normal/unsaturated	
16:1 w7c alcohol	0.4
16:2 DMA	0.9
17:1 w7c	0.7
17:1 w6c	1.4
18:3 w3c	1.0
18:2 w6c	4.2
18:1 w9c	7.7
18:1 w7c	1.7
18:1 w9c DMA	3.7
18:1 w7c DMA	1.2
19:1 w6c	0.6
22:1 w9c	1.7
	Total = 24.9 %
	Grand total = 100 %

^a^Carbon number for double bond positions (w) relative to the methyl carbon end of the hydrocarbon chain; DMA refers to dimethyl acetal group occurring at the carboxyl (#1 carbon) end

### Taxonomic alignment of strain SLSR-1

**Fig. 5:**
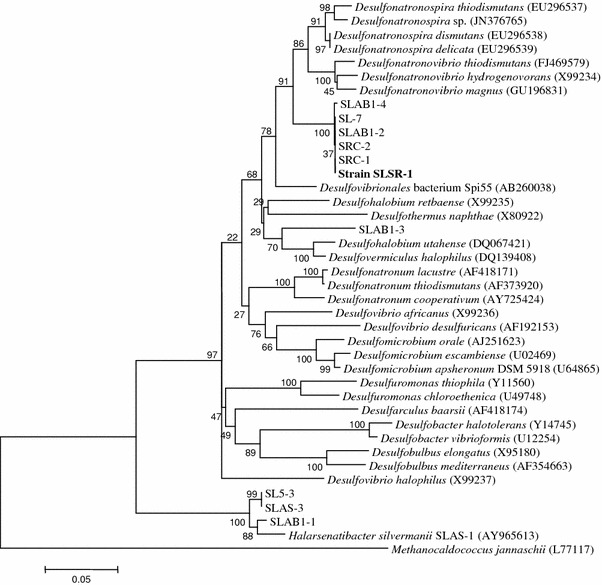
Neighbor-joining tree of 16S rRNA genes showing the phylogenetic position of strain SLSR-1 and other amplicon sequences obtained from Searles Lake (prefix *SL*). Genbank accession numbers are included next to the corresponding species names

Phylogenetic analysis of the 16S rRNA gene sequences shows that strain SLSR-1 and other sequences obtained from Searles Lake sediments (SLAB, SL, and SRC) form a distinct clade in the family *Desulfohalobiaceae* near the root of the genus *Desulfonatronovibrio* (Fig. 5). Members of this family of sulfidogenic haloakaliphiles exhibit diverse substrate affinities and salt tolerances. *Desulfonatronovibrio hydrogenovorans* is a sulfate reducer that can use H_2_ or formate as electron donors for reduction of sulfate, sulfite, or thiosulfate (Zhilina et al. 1997). Other members of this family include the genus *Desulfonatronum* which in addition to their use of H_2_ or formate, can use ethanol or in one case lactate (Pikuta et al. 2003; Zhilina et al. 2005). Members of the genus *Desulfonatrospira* have a more versatile metabolism, in that they can use H_2_ for the reduction of sulfate or sulfite along with electron donors like lactate and alcohols, but can also ferment pyruvate or grow chemoautotrophically via disproportionation of either sulfite or thiosulfate (Sorokin et al. 2008). The moderate halophile *Desulfovermiculus halophilus* not only has metabolic versatility in being a chemo-organotroph with a broad array of organic electron donors, but also harbors the ability to grow as an autotroph on H_2_ + CO_2_ (Beliakova et al. 2006). Another denizen of anoxic soda lake mud worth mentioning is *Desulfurispira natronophila*. But while this bacterium is a moderate haloalkaliphile that employs a diverse array of organic electron donors to achieve growth, it uses elemental sulfur or arsenate as electron acceptors but cannot use sulfate, thiosulfate, or nitrate. Indeed, it belongs to the family *Crysiogenetes* rather than the *Desulfohalobiaceae* (Sorokin and Muyzer 2010).

**Fig. 6:**
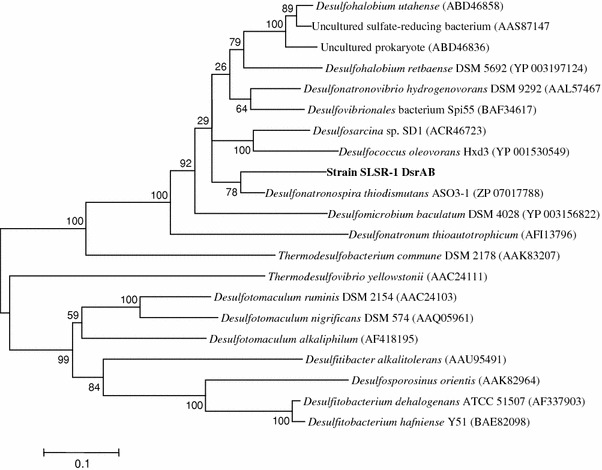
Maximum likelihood tree of predicted amino acid translation of the *dsrAB* genes showing the phylogenetic position of strain SLSR-1. Genbank accession numbers are included next to the corresponding species names

Despite being displayed in Fig. 5 at the base of the *Desulfonatronovibrio* genus, *Desulfonatronovibrio hydrogenovorans* shows only 90 % homology with strain SLSR-1 based on 16S rRNA gene sequence BLAST alignments. The closest phylogenetic relatives of strain SLSR-1 are from the genus *Desulfonatronospira*, with highest homologies for *D.spira thiodismutans* (95 %), *D.spira delicata* (94 %) and *D.spira dismutans* (94 %) (Fig. 5). The DsrAB phylogeny also places SLSR-1 within the *Desulfohalobiaceae* (Fig. 6), being most similar to *Desulfonatronospira thiodismutans* (Sorokin et al. 2008). A similar case can be made for the respiratory arsenate reductase of strain SLSR-1 (see below). But there are substrate disparities between strain SLSR-1 and these diverse haloalkaliphiles. As mentioned above, chemoautotrophic growth via disproportionation of sulfite or thiosulfate is a distinguishing characteristic of *Desulfonatronospira thiodismutans* and *Desulfonatronospira delicata* in addition to heterotrophic growth by oxidation of low-molecular-weight organics (e.g., lactate, ethanol, butanol) linked to respiratory reduction of sulfate, thiosulfate, or sulfite (Table 2; Sorokin et al. 2008). The ability to metabolize H_2_ was observed for *Desulfonatronum thiodismutans*, *Desulfonatronum cooperativum*, *Desulfonatronovibrio hydrogenovorans* (Table 2), and *Desulfurispira natronophila* (Sorokin and Muyzer 2010) However, strain SLSR-1 was not capable of H_2_ metabolism, and although it could grow via dissimilatory reduction of thiosulfate or sulfite, it did not display a clear capacity for chemoautotrophic growth via disproportionation of these sulfur compounds. Moreover, although strain SLSR-1 could use pyruvate as an electron donor, it could not grow via pyruvate fermentation in the absence of an electron acceptor, nor could it employ acetate as an electron donor for growth on sulfate. Finally, strain SLSR-1 is clearly morphologically distinct from the *Desulfonatronospira* by having a distinct vibroid rather than a spiraled shape, which when taken together with its lower G + C abundance (e.g., 45.1 vs. 50.4 %) argues for its taxonomic classification in a separate genus.

Strain SLSR-1did exhibit a mode of sustained growth with sulfide as the electron donor and nitrate as the electron acceptor which would suggest a capacity for chemolithotrophy. However, when the sulfide/arsenate couple was tested cell lines could not be sustained after one or two transfers, indicating a requirement for a carbon skeleton (e.g., lactate, acetate) rather than via autotrophic fixation of inorganic carbon. Nonetheless, nitrate- or arsenate-linked lithotrophic oxidation of sulfide is analogous to modes of chemoautotrophy displayed by two isolates from Mono Lake, *Alkalilimnicola ehrlichii* strain MLHE-1 and strain MLMS-1 (Oremland et al. 2002; Hoeft et al. 2004, 2007), and by *Halarsenatibacter silvermanii* from Searles Lake (Oremland et al. 2005).

**Fig. 7:**
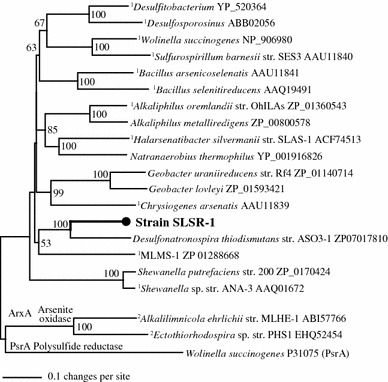
Phylogenetic relationship of the ArrA sequence of strain SLSR-1 in relationship to the ArrA enzymes of other known arsenate respiring bacteria or predicted ArrA identified in sequenced microbial genomes. The bootstrap values 50 % and greater are indicated at the corresponding nodes. Superscript 1 and 2 correspond to organisms known to respire arsenate or oxidize arsenite, respectively

Phylogenetic analysis of the predicted protein of the *arrA*-like gene sequence of SLSR-1 revealed that it is part of the ArrA, arsenate respiratory reductase clade of the DMSO reductase family of molybdenum enzymes (Saltikov and Newman 2003). The SLSR-1 partial ArrA was most similar to an ArrA-like encoding gene found in the genome of *Desulfonatronospira thiodismutans* (71 and 82 % amino acid identity and similarity, respectively) although it is not known if this organism can grow via dissimilatory arsenate reduction (Fig. 7). Strain SLSR-1 ArrA formed a cluster with ArrAs of *D.spira thiodismutans* and the Mono Lake sulfide-oxidizing arsenate reducer, MLMS-1. Additional partial *arrA* sequences from uncultured bacteria previously retrieved from Searles Lake using a different primer set (Kulp et al. 2006) shared only 70–80 % amino sequence similarities to the corresponding regions of the SLSR-1 ArrA. These observations indicated that the strain SLSR-1 arsenate respiratory reductase is most likely a unique member of the ArrA clade. These collective considerations argue that strain SLSR-1 represents the first described member species of a new genus of extremely haloalkaliphilic sulfate-respiring bacteria, for which we propose the name *Desulfohalophilus alkaliarsenatis* gen. nov., sp. nov.

### Presence of SLSR-1-like sequences in Searles Lake

Although strain SLSR-1 was isolated from a stable enrichment culture from Searles Lake, its significance to the Searles Lake ecosystem is unclear. DGGE analysis indicates that sequences closely related to SLSR-1 are present in sediments of Searles Lake and are predominant at 7.5 cm depth in the sediments. The presence of strain SLSR-1 relatives in Searles Lake sediments is supported by our previous finding of sequences highly related to SLSR-1 (Fig. 5; Kulp et al. 2006). However, the *arrA* gene from strain SLSR-1 was distinct from those found in Searles Lake samples. Thus, we cannot directly confirm that this isolate is a dominant member of the Searles Lake microbial community. Nonetheless, the physiology of this isolate, i.e. its capacity to reduce As(V) under conditions restrictive for sulfate reduction, is likely a common mechanism in Searles Lake and other As(V) and SO_4_^2−^ containing environments.

## Discussion

Strain SLSR-1 demonstrated slow but obvious growth as an anaerobic heterotroph when cultivated in defined mineral medium with sulfate or in combinations of sulfate plus arsenate serving as electron acceptors under conditions of moderate hypersalinity (at an *a*_w_ of 0.9, Fig. 3). We noted that growth on arsenate required the presence of sulfate. This could have been due to the latter anion serving as a nutritional source of sulfur in this chemically defined medium which lacked any complex nutritional supplements (e.g., yeast extract, peptone) and contained only a small quantity of cysteine serving as a reductant. When both arsenate and sulfate were present at equimolar concentrations, dissimilatory reduction of the former preceded that of the latter (Fig. 3b, c), a condition first noted for *Desulfotomaculum auripigmentum* (renamed: *Desulfosporosinus auripigmenti*) (Newman et al. 1997a), and explained by the much greater energy yield associated with respiration of arsenate as opposed to sulfate (Newman et al. 1997b). Supplementing the medium with 0.2 % yeast extract and incubating at a salinity closer to optimal (*a*_w_ = 0.86) decreased doubling times from 82.5 to 18.4 h, and increasing μ from 0.008 to 0.038 h^−1^.

Despite the extraordinarily high concentration of sulfate in the brines of Searles Lake (0.730 molal; roughly equivalent to 1 M), in situ sulfate-reduction was undetectable in its sediments (Oremland et al. 2005; Kulp et al. 2006, 2007). Sulfate-reduction by strain SLSR-1 was strongly inhibited by the imposed salinity gradients (Fig. 4a), being almost completely eliminated at a salinity (~330 g/L; *a*_w_ = 0.79) that approached the actual strength of the lake’s brine (*a*_w_ = 0.71; Table 1). Hence, these results are in general accordance with the original observations of Oren (1999a) with regard to the bioenergetic-constraints upon sulfate-reduction at high salinities. The survival of strain SLSR-1 under such ambient conditions in Searles Lake is assured by its ability to grow via respiration of the abundant arsenate ions in the brine (~4 mM; Oremland et al. 2005) in lieu of sulfate. Arsenate reduction by strain SLSR-1 was impeded comparatively modestly (~50 %) at the highest salinity tested (Fig. 4a).

The natural abundance of borate ions in the brinewater of Searles Lake (0.43 molal) was postulated as a selective cause of inhibition of sulfate-reduction in its sediments (Kulp et al. 2007). High borate concentrations strongly inhibited sulfate-reduction by strain SLSR-1 (93 % inhibition), while its effect on arsenate-reduction was less pronounced (78 % inhibition) (Fig. 4c) leaving this variable as a contributing complication to the original salinity conundrum. Faced with the “double whammy” of Searles Lake brine’s low water activity combined with its high borate concentrations, it appears that strain SLAS-1 is operating in situ at the margin of its physiological survival range. Nonetheless, vigorous in situ ^73^As(V)-reduction was measured in the anoxic, shallow sediments of Searles Lake that lay beneath its thick salt crust and overlying brine (Kulp et al. 2006). The results prove that the bacterial community, if not strain SLSR-1 itself, is functional under these extreme conditions. Examples of poly-extremophiles, namely the ability to tolerate multiple severe environmental stresses, can be found in other microorganisms isolated from evaporative soda lakes (Bowers and Wiegel 2011).

Low but detectable free sulfide (~0.1–0.3 mM) is prevalent within the anoxic sediment lying below the dense brinewater of Searles Lake (Oremland et al. 2005; Kulp et al. 2006). In the absence of sulfate-reduction, the origin of the sulfide could arise from the dissimilatory reduction or disproportionation of less oxidized intermediates of the sulfur cycle such as thiosulfate, sulfite, or elemental sulfur. The bacteria that carry out these types of sulfidogenesis occur commonly in soda lakes (Sorokin et al. 2008, 2011a; Sorokin and Muyzer 2010), as do the bacteria that regenerate these substances by oxidative processes (Sorokin et al. 2011b). Strain SLSR-1 was capable of growth upon thiosulfate and sulfite via dissimilatory reduction, although notably not elemental sulfur, as were its closest phylogenetic relatives listed in Table 2. *Desulfurispira natronophila* can grow on sulfur in lieu of sulfate, thiosulfate, or sulfite (Table 2), as can *Halarsenatibacter silvermanii* an arsenate-respirer isolated from Searles Lake (Switzer Blum et al. 2009). Collectively, a sediment flora consisting of substrate affinities represented by these haloalkaliphiles would assure modes of in situ sulfide production via intermediates of the sulfur cycle. Supporting this is the detection of 16S rDNA amplicons of strains taxonomically related to strain SLSR-1 from extracted DNA from Searles Lake mud (Fig. 5). Yet that leaves unanswered the question of how intermediates of the sulfur cycle arose in the first place without the occurrence of some initial sulfate-reduction.

One possibility is that the free sulfide currently detected in the lake’s sediments could have arisen from the degradation of organic sulfur compounds, such as the common osmolyte dimethylsulfoniopropionate, and perhaps from sulfur-containing proteins as well. The question of the origin of the sulfide detected in Searles Lake is ecologically relevant because both strain SLSR-1 and *H. silvermanii* also have the ability to oxidize sulfide using either arsenate or nitrate as the oxidant (Table 2; Oremland et al. 2005; Switzer Blum et al. 2009).

The search for extant microbial life in the Solar System beyond Earth’s orbit holds the likelihood of someday encountering and testing highly dense brines formed either by cryo-concentration (Europa) or evapo-concentration (Mars). Planetary scientists and geochemists employ *a*_w_, rather than salinity, as a means to describe the density of such brines. Specifically, in the case of Mars the residual fluvial salt deposits encountered by the Rover Opportunity at Meridiani Planum have been proposed to be derived from brines having such strong ionic strengths as to severely impede or even preclude the possibility of life. Tosca et al. (2008) calculated *a*_w_ values of 0.78–0.86 of the brines that once flowed over these sites, roughly 3.0 billion years ago (Andrews-Hanna et al. 2007). Strain SLSR-1 can clearly grow at these low water activities (Table 1; Fig. 4), and in situ As(V)-reductase activity occurs in the sediments of Searles Lake at *a*_w_ = 0.71 (Kulp et al. 2006). Hence, the consideration of salinity per se as an absolute impediment to anaerobic microbial life at these *a*_w_ values can be challenged, although the brine’s highly acidic rather than alkaline nature would add another strong physiological constraint. Another severe physiological constraint would include considerations of chaotropicity (high MgCl_2_ content; Hallsworth et al. 2007). However, the gypsum veins of the early Noachian age recently encountered by the Rover Opportunity at the Cape York site (Endeavor Crater) gave “evidence for relatively dilute [*a*_w_ = ~0.98] water at moderate temperature, perhaps supporting locally and transiently habitable environments” (Squyres et al. 2012). Such a scenario could conceivably allow time for the adaptation to and natural selection of a microbial flora able to tolerate the extreme conditions that subsequently ensued over the Meridiani Planum of the late Noachian and early Hesperian epochs of Mars. Regardless of these events, adopting a general usage of *a*_w_ within the extremophile community would make it better conversant with planetary scientists searching for extant (or extinct) life at the margins of known terrestrial physiology encountered in possible biomes lying beyond Earth.

### Description of *Desulfohalophilus*, gen. nov.

De.sul.fo.ha.lo’phi.lus. L. pref. de-, from; L. n. sulfur, sulfur; N.L. pref. desulfo-, desulfuricating (prefix used to characterize a dissimilatory sulfate-reducing prokaryote); Gr. n. hals halos, salt; N.L. adj. philus (from Gr. adj. philos -ê -on), friend, loving; N.L. masc. n. *Desulfohalophilus*, sulfate-reducing salt-loving bacterium; Gram negative motile vibrios. Obligately anaerobic with a respiratory metabolism of dissimilatory reduction of sulfate. Chemoorganotroph, incompletely oxidizes electron donors like lactate and pyruvate. Alkaliphilic and halophilic. Habitats are soda lakes. Belongs to the class deltaproteobacteria. The type species is *Desulfohalophilus alkaliarsenatis*.

### Description of *Desulfohalophilus alkaliarsenatis* gen. nov., sp. nov.

Motile vibrio with one or two polar flagella. Cells are 0.5 × 3.0 μm, occur singly or in pairs. Multiplication is by binary fission, and cells have a Gram negative cell wall structure. Strictly anaerobic, growth is chemohetereotrophic or chemoautotrophic. Slow growth occurs in defined mineral medium, but is stimulated by the inclusion of yeast extract. Utilizes lactate or pyruvate as electron donors when grown with sulfate or arsenate as the electron acceptor, the products include acetate, sulfide or arsenite. Other electron acceptors supporting heterotrophic growth include nitrate, sulfite and thiosulfate. Chemoautotrophic growth occurs with sulfide as the electron donor and nitrate as the electron acceptor. Lithotrophic oxidation of sulfide with arsenate, but requires a carbon skeleton (e.g., lactate) for growth. Obligate alkaliphile that does not grow at pH below 7.75 or above 9.75 and has an optimum at 9.25. Salinity range for growth ranges from 55 to 330 g/L (*a*_w_ = 0.96–0.8) with an optimum at 200 g L^−1^ (0.84 *a*_w_). The optimum temperature for growth is 44 °C and growth occurs over the range of 20–55 °C. The G + C content is 45.1 mol% (as determined by the thermal denaturation method). Habitat: bottom deposits of alkaline athlassic and extremely hypersaline soda lakes. The type strain is strain SLSR-1, which was isolated from the sediments of Searles Lake, located in the Mojave Desert of southeastern California, USA. The strain has been deposited in the American Type Culture Collection (accession number BAA-2432) and the DSMZ (DSM accession number 25765)
